# Acceptance of the COVID-19 Vaccine by Prisoners and Staff in Spanish Prisons

**DOI:** 10.3390/vaccines11101547

**Published:** 2023-09-29

**Authors:** Nancy Vicente-Alcalde, Sorina Madalina Sferle, Carlos Franco-Paredes, José Tuells

**Affiliations:** 1 Penitentiary Center Alicante II, Carretera N-330, Km. 66, 03400 Villena, Spain; 2 Instituto Universitario de Matemática Multidisciplinar, Universitat Politècnica de València, Camino de Vera, s/n, 46022 Valencia, Spain; smsferle@doctor.upv.es; 3 Department of Microbiology, Immunology and Pathology, Children’s Hospital of Mexico Federico Gomez, Dr. Márquez 162, Ciudad de Mexico 06720, Mexico; carlos.franco.paredes@gmail.com; 4 Department of Community Nursing, Preventive Medicine and Public Health and History of Science, University of Alicante, San Vicente del Raspeig, 03690 Alicante, Spain; tuells@ua.es

**Keywords:** COVID-19, vaccine, acceptance, prisons, public health

## Abstract

The COVID-19 vaccination of prisoners and prison staff represents a public health intervention to reduce the impact of the pandemic in conglomerate settings. In Spanish prisons, the road map of the Ministry of Health was followed to protect the population at risk. We conducted a cross-sectional study to assess the acceptance of COVID-19 vaccination by prisoners and prison staff in a prison in Alicante, Spain. We analyzed data obtained through a standardized, self-administered, and anonymous questionnaire; 1016 prisoners and 288 prison staff responded to the survey. The majority of inmates and staff reported no history of symptomatic COVID-19, 90.15% and 91.66%, respectively. Respondents reported that 88.72% agreed to be vaccinated and 89.64% would recommend the vaccine to others. Approximately 89% believe that the benefit of getting vaccinated against COVID-19 is greater than the risk, and 70.55% reported that vaccination should be mandatory for inmates and staff to participate in some activities. The acceptance of COVID-19 vaccination among prisoners and prison staff is high in a Provincial Prison in Spain. Elevated acceptance of COVID-19 vaccination in prisons is a major factor in public health intervention and vaccine equity.

## 1. Introduction

Prisons are dynamic ecological environments in which prisoners, staff, officers, and visitors transit daily between the prison and the larger community [1]. Incarcerated individuals live in confined spaces, often with poor ventilation and overcrowding. These factors are conducive to the spread and amplification of infectious diseases inside and outside prisons, making carceral settings more vulnerable to outbreaks of coronavirus disease (COVID-19) [2,3]. The Ministry of Health in Spain paid close attention to the COVID-19 pandemic by establishing guidelines to address the vulnerability of prisoners and prison staff [4,5,6]. As a result, the COVID-19 vaccination of prisoners and prison staff was considered a crucial public health intervention [6,7,8,9].

Vaccination coverage against COVID-19 in prisons (incarcerated individuals and staff) in certain countries reached levels similar to that of the general population even during periods of limited vaccine supply [10,11,12]. However, globally, there is evidence that vaccination against COVID-19 among inmates and prison staff was not considered a public health priority [10,11,12].

Vaccination in Spanish prisons began in April 2021 [13], responding to the national strategy to deploy the COVID-19 vaccination to high-risk populations [14] and following the World Health Organization (WHO) vaccination against COVID-19 guidelines [15]. COVID-19 vaccination in Spain was offered on a voluntary basis [16]. During the initial deployment of the COVID-19 vaccination, the prison population in Spain amounted to more than 51,000 people and the number of workers laboring inside penitentiary institutions was 28,460, corresponding to a ratio of 1.9 prisoner/worker, which places Spain above the European average for this proportion [17,18].

The availability and wide deployment of a newly introduced vaccine to protect against a life-threatening infection does not imply an immediate acceptance of vaccination by the general population. According to the 5Cs model, vaccine acceptability is contingent upon trust, risk perception, information-seeking behavior, and the willingness to protect others [19]. The effect described as “Pandemic Public Health Paradox” associates the acceptance of a vaccine with the influence of the media rather than with the epidemiological dynamics of the disease [20]. Indeed, there are different factors that can pose an obstacle to vaccine acceptance, including ease of access to reach vaccination sites, geographical location, accessibility, availability, health system factors, and the affordability of vaccines, as well as cultural, psychological, emotional, cognitive, social, and political factors [21].

The objective of this study was to ascertain the level of acceptance of vaccination against COVID-19 in Spanish prisoners and prison workers.

## 2. Materials and Methods

### 2.1. Design and Participants

We conducted a cross-sectional study to assess the degree of COVID-19 vaccine acceptance in prisoners of the Alicante II-Villena Penitentiary Center and Spanish prison workers. The exclusion criteria were refusal to participate in the study or answering the survey inappropriately or with information not requested for the study.

### 2.2. Tools

We used a self-administered, anonymous, and standardized questionnaire to evaluate the acceptance of the COVID-19 vaccine in prisoners and prison staff. The questionnaire, designed “Ad hoc” for this project, consisted of a total of 36 questions, including sociodemographic variables, some of them posed with multiple answers and others on a nominal scale. Its structure was divided into three sections: (i) baseline socio-demographic questionnaire (sex, age, origin, marital status, number of children) and personal health variables (items 1 to 5: suffering from chronic disease, smoking, alcohol, psychotropic drugs); (ii) COVID-19 variables (items 6 to 16: suffering from COVID-19 disease, contagion in a prison environment, need for quarantine, death of a family member or friend due to COVID-19, PCR performance, PCR results); (iii) acceptance of the COVID-19 vaccine (items 17 to 36: vaccination, previous influenza vaccination, adverse effects of vaccination, confidence in the recommendations of the health authorities, reasons for acceptance or rejection of the vaccine). We developed the questions in this survey based on a review of the medical literature and approval by consensus from all researchers.

### 2.3. Study

At the time of the study, the Alicante II Penitentiary Center had 1037 prisoners and 312 workers. We included 1304 participants (1016 (98%) prisoners and 288 (92.3%) workers) who responded to a voluntarily self-administered survey during the months of April to June 2021. To use an appropriate sampling frame, it was decided to recruit participants through the prison health team, providing them with information about the project, requests to participate by obtaining an informed consent, and the actual survey.

All prisoners and prison staff who showed a willingness to participate and who met the inclusion criteria were included. Sample selection was carried out in a “non-probabilistic” manner, using convenience sampling.

### 2.4. Statistical Analysis

We conducted a descriptive univariate analysis on all the variables. For categorical variables we calculated frequencies and percentages, and continuous variables, such as age, were categorized by age group. We also carried out a descriptive bivariate analysis. For pairs of categorical variables, we generated contingency tables and calculated the corresponding column percentages. In such cases, we employed the Chi-squared test to assess the association between these variables. To assess factors influencing the response to questions related to COVID-19 vaccine acceptance, logistic regression analysis was performed for cases in which the response variable had two categories and multinomial logistic regression analysis for those with more than two categories. To determine whether each predictor variable has a statistically significant effect on the response variable, we obtained *p*-values using the Wald test. We exponentiated the coefficients to better interpret the results obtained, thus providing odds ratios (OR) for the logistic model and relative risk ratios (RRR) for the multinomial model, with their respective 95% confidence intervals (CI95%). Before performing these analyses, we determined whether there was an association or dependence between the categorical variables using the chi-squared test or Fisher’s exact test, when the sample size of the resulting contingency tables was small. We set the significance level at α=0.05. All analyses were performed using RStudio, specifically version 4.0.2 of the open-source software.

### 2.5. Ethical Considerations

The study was approved by the General Secretary of Penitentiary Institutions, Sub-directorate General of Institutional Relations and Territorial Coordination (number 395714). Participation in the survey was conducted anonymously and voluntarily, and the analysis of the questionnaire was completely confidential, ensuring the privacy of the data of all respondents. For this study, we followed the ethical principles for medical research involving human subjects set out in the Declaration of Helsinki and EU Regulation 134 2016/679 (GDPR) regarding the processing of personal data.

## 3. Results

### 3.1. Population Description

We analyzed data obtained from 1304 surveys, of which 1016 were from incarcerated individuals. Of these, most were Spanish citizens (92.61%), and most were men (89.96%), married (59.84%), had children (70.37%), and were between 40 and 60 years of age (45.67%). Approximately 98% of prisoners in the Alicante Penitentiary participated in the survey. Just over half of the prisoners said they were not suffering from any chronic medical disease (65.45%), most were smokers (75%), and most did not consume alcohol (78.24%) (Table 1). However, 33.27% reported the active use of psychotropic drugs. Almost all prison staff who responded to the survey were Spanish citizens (99.31%), 51.04% were men, 44% were married, 61.8% had children, and the ages ranged from 40 to 60 years old (71.88%). From the *p*-values obtained using the chi-squared test, we can conclude that there was a relationship between each of the categorical variables (those shown in the first column) and the response variable composed of the prisoners and prison staff categories. In other words, the test indicates that demographic characteristics are associated with differences in professionalism among participants.

### 3.2. Variables Related to COVID-19 Disease

At the time of conducting the study, a high percentage of participants had not suffered from symptomatic COVID-19 infection (90.15% of prisoners and 91.66% of workers) (Table 2). However, 69.68% of prisoners had been placed in quarantine due to close contact with a confirmed case and 18.89% reported the death of a family member or friend due to COVID-19. Approximately 67.42% had to undergo PCR testing or antigen testing to detect SARS-CoV-2. Results were negative in 85.40% of tests performed. Just 32.48% of prisoners reported limiting their contacts or social relationships with others during the pandemic.

Among prison staff, 24.30% had been placed in quarantine due to contact with a confirmed case and 18.40% reported the death of a family member or friend due to COVID-19. Over half of the survey respondents (52.08%) underwent PCR testing or antigen testing to detect SARS-CoV-2. Results were negative in 92% of tests performed. Almost all prison staff (98.95%) reported limiting their social interactions during the pandemic.

With respect to the *p*-values, we observed that having or not having COVID-19 disease (0.5113), and whether or not an acquaintance had died from this infectious disease (0.9169), was not related to the response variable. In practical terms, this means that there is no statistically significant association between having had COVID-19 or having an acquaintance who died and professionalism among inmates and prison staff. However, the variables related to having undergone quarantine (<0.001), having taken a diagnostic test (<0.001) and having reduced social interactions (<0.001) were related to the response variable. Therefore, these do depend on whether one is a prisoner or prison staff; for example, with respect to the last variable there is a clear difference in that prisoners are negligent in continuing to have social contact.

### 3.3. Variables Related to COVID-19 Vaccine Acceptance and Attitude towards Vaccination

As depicted in Table 3, 88.72% of the prisoners and prison staff who responded to the survey reported that they would accept COVID-19 vaccination, and 89.64% would recommend the vaccine to others. However, recommending the vaccine to children was reported only by 67.71% of study participants. Approximately 89% believe that the benefit of undergoing COVID-19 vaccination is greater than the risk, and 70.55% reported that vaccination should be mandatory for prisoners and staff to participate in some activities. It is important to note that vaccine acceptance for the seasonal influenza vaccine during the 2019–2020 season was low, resulting in 86.71 prisoners and 44.44% prison staff not receiving the influenza vaccination.

Most prisoners who were vaccinated (97.24%) received Jcovden^®^ and prison staff who were vaccinated (88.89%) received mostly Vaxzervria^®^.

We can see that all questions were associated with the response variable, since the *p*-values were below the significance level (*p*-values < 0.05). All of these significant values collectively point to the fact that there are pronounced differences between inmates and prison staff in their attitudes and behaviors related to COVID-19 vaccines.

### 3.4. Relative Risk Ratios from the Multinomial Logistic Regression Model and Odds Ratios from the Logistic Regression Model by Age, Sex and Comorbidity

Important factors to evaluate for our study are sex, gender and comorbidity. We can see that in most cases all three are related to the questions based on COVID-19 vaccine acceptance. For a more comprehensive interpretation of these relationships, we focused on the RRRs and ORs of those statistically significant cases, i.e., when the *p*-values were less than 0.05 (Table 4).

Males were 67% less likely to choose “yes” (getting vaccinated) compared to females. The *p*-value (*p* = 0.0015) indicates that the difference is statistically significant, so there is strong evidence that being male is associated with a lower probability of choosing to be vaccinated.

The extreme values associated with the “>60” category of the age variable were due to the presence of zero cell counts, since all participants in this category responded affirmatively, which caused calculation problems.

Being in the “40–<60” age group did not significantly affect the likelihood of choosing to be vaccinated or waiting a few more months; however, individuals aged over 60 were more likely to choose “yes” compared to those in the “18–<40” age group.

Individuals with a chronic disease were 1.86 times more likely to be willing to be vaccinated than those without a chronic disease, where the *p*-value = 0.008 indicates that the association between having a chronic disease and being willing to be vaccinated is statistically significant. Having chronic diseases appears to be associated with an increased likelihood of answering “yes” to all questions related to COVID-19 vaccine acceptance, except in the case of agreeing that it would seem appropriate to be required to be vaccinated for certain activities, where the difference may not be statistically significant (*p*-value = 0.06).

There was no statistical difference in the willingness to vaccinate their children between males and females; however, there were statistically significant differences in preferences for waiting periods, since males were significantly less likely (0.21 times and *p*-value < 0.001) to prefer waiting a few more months compared to females.

Age appeared to be significantly associated with preferences regarding waiting times for vaccination among children. Individuals aged “40–<60” were significantly less likely to prefer waiting a few more months (0.29 times and *p*-value < 0.001) or waiting for others to try the vaccine (0.48 times and *p*-values = 0.002) before vaccinating their children compared to those aged “18–<40”. However, the age group “>60” showed no significant differences in these preferences compared to the “18–<40” group.

The OR = 0.36 with *p*-value = 0.0015 suggests that gender had a significant influence on the recommendation of the COVID-19 vaccine, with females being more likely to recommend it than males.

The analysis suggests that gender is strongly associated (*p*-value < 0.001) with the perception of the benefit of COVID-19 vaccination. Females were more likely to believe that being vaccinated is beneficial. The OR = 0.71 (*p*-value = 0.035) indicates that gender appears to have a significant effect on the perception of whether vaccination should be required for certain activities, with males being less likely to agree with such a requirement compared to females.

Individuals in the “40–<60” age group were more likely, compared to those in the “18–< 40” age group, to hold the belief that the benefits of vaccination outweigh those of not vaccinating, as indicated by the OR of 1.76 (*p*-value = 0.0015). The odds of agreeing that it would seem appropriate to be required to be vaccinated for certain activities were 6.54 times the odds for the “18–<40” age group. However, the difference in the “40–<60” age group may not be statistically significant.

Finally, being female, being in the “40–<60” or “>60” age groups, and having a chronic disease were associated with higher odds of having received the flu vaccine during the 2019–2020 vaccination campaign. These associations were statistically significant.

### 3.5. Reasons for COVID-19 Vaccines Acceptance or Refusal

**Figure 1:**
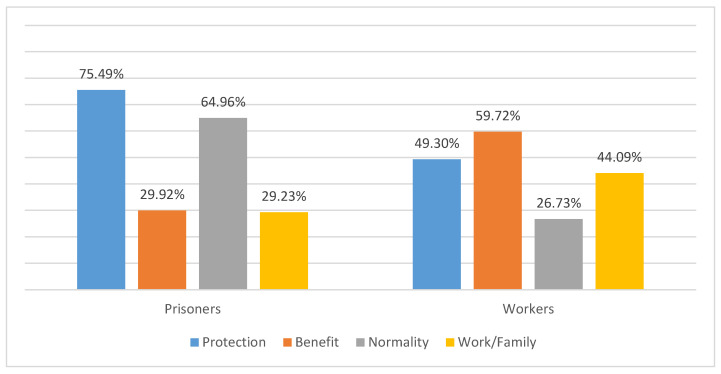
Acceptance of COVID-19 vaccines in prisoners and prison staff of the Alicante II Penitentiary Center.

The reasons for the acceptance or rejection of the COVID-19 vaccine are shown in Figure 1. The main reasons for accepting a COVID-19 vaccine were associated with individual protection against the disease. Likewise, the reasons for acceptance referred to by most prisoners and workers were: (1) Protection of oneself. (2) The benefits of vaccines. (3) Return to normal. (4) For work reasons or protection towards the family. Regarding prisoners, self-protection and a return to normality predominated over the benefits of the vaccine and work or family protection reasons. On the other hand, in the case of prison staff, the majority reported accepting COVID-19 vaccination for all four stated reasons and benefits.

### 3.6. Beliefs and Occurrence of Adverse Effects after the Administration of the COVID-19 Vaccine

Table 5 shows that both prisoners (83.17%) and prison staff (66.67%) reported that COVID-19 vaccination is not associated with more adverse effects than other vaccines, but on the contrary, they believed that there were unknown adverse effects (71.06% prisoners and 57.99% prison staff). Approximately 61.11% reported that the accelerated pace of vaccine production had no effect in reducing its safety and 81.82% reported to trust recommendations made by public health authorities. Regarding the adverse effects suffered by the administration of the vaccine, 56.11% reported having suffered them, with prison staff reporting a higher frequency than prisoners; 69.92% and 52.53%, respectively. The most frequent adverse effects reported were pain at the injection site (69.91%), generalized muscle aches (59.45%) and chills (57.59%). In addition, the column associated with the *p*-values suggests that there was a statistically significant association between each variable (questions) and the response variable. This result implies that the groups (prisoners and prison staff) do not have the same views about adverse effects following the COVID-19 vaccination.

## 4. Discussion

The COVID-19 pandemic substantially impacted congregate facilities such as jails and prisons. Correctional facilities concentrate large populations of incarcerated individuals, and many of them have underlying chronic medical conditions that predispose them to negative clinical outcomes during outbreaks of highly transmissible infectious pathogens. As such, it is crucial to implement interventions to reduce the impact of infectious outbreaks among prison staff and incarcerated individuals from a medical and public health perspective. In this regard, vaccination against COVID-19 offers the possibility to reduce medical complications associated with SARS-CoV-2 infections for incarcerated individuals and staff working in correctional facilities.

To our knowledge, this study is the first conducted on the acceptance of the COVID-19 vaccine among incarcerated individuals and prison staff in Spanish prisons. Most reports are from the United States, the country with the highest number of incarcerated individuals worldwide. Our results are similar to those reported in other carceral settings [5,11,12,22,23,24]. According to studies conducted in carceral settings in the U.S. the incidence of COVID-19 infections in prisons was identified in some jails and prisons to be 5.5 times higher than in the community [4,25,26]. Prior to the start of the COVID-19 vaccination campaign in prisons, the pandemic forced the adoption of a series of restrictive measures to prevent infections in prisons [27]. However, there is evidence to suggest that, in the prison settings, with the implementation of COVID-19 vaccinations, the number of cases and fatalities due to COVID-19 were reduced substantially, demonstrating the effectiveness of COVID-19 vaccination in incarcerated individuals and prison staff [22,27].

Ismail, N et al. in their review study concluded that conducting more empirical research on the acceptability of COVID-19 vaccination would help to reduce the impact of COVID-19 on the prison population, prevent community transmission, improve vaccine acceptance in prisons, encourage political accountability and inform future decision-making [10].

Our study highlights the high acceptance of COVID-19 vaccination by Spanish prisoners and prison staff, which almost reached the levels of vaccination coverage in the general population, as it has been reported in the United Kingdom and Wales [28]. Among prisons in Catalonia, full vaccination against COVID-19 was achieved in 72.9% of their prison populations, in contrast to that achieved in Alicante, of 95.39%. There was a variability of COVID-19 vaccination acceptance, leading to highly susceptible populations being unprotected in other settings. For example, studies carried out in jails and prisons in the United States show lower vaccine acceptance, similar to the overall low vaccination acceptance in the larger community [11,12,22,24,29].

Our study shows that at the time of administration of the COVID-19 vaccine, acceptance was higher than that reported by prisoners initially. Other studies have shown that among prisoners who initially declined the first dose of the vaccine, a significant fraction accepted it when it was reoffered, so vaccine hesitancy is not permanent [11,22,24]. According to our study, one of the main reasons for accepting the COVID-19 vaccine among Spanish prisoners was the desire to return to normality after the institution of social distancing and restrictive measures, which highlights the trust of this population in recommendations made by health authorities. This last point turned out to be a determining factor in the acceptance or not of the vaccine against COVID-19, as can be seen in other, similar studies, in which a lack of trust in health personnel was a key factor in accepting or refusing vaccination [12,30,31].

On the other hand, we observed that vaccination among Spanish prison staff was slightly lower than that of prisoners. This fact was also reflected in studies carried out in the U.S. [11,22,32]. Among our findings, nearly half of prison staff believe that the accelerated pace of vaccine production reduced their safety; this belief was also reported as the main reason for COVID-19 vaccination refusal among prison staff in Massachusetts [29].

Currently, in Spanish prisons, high vaccination coverage against COVID-19, along with immunity generated from natural infections, allows us to consider that the majority of the prison population is currently protected against COVID-19 [33,34].

## 5. Conclusions

The SARS-CoV-2 pandemic has highlighted the vulnerability of incarcerated individuals and prison staff. The high levels of acceptance and vaccination against COVID-19 in Spanish prisons are a measure of responses to public health needs of incarcerated individuals and prison staff. The results of this study demonstrate that vaccine acceptability was high in a prison system during a public health emergency such during a pandemic. While we cannot extrapolate these results to other settings, to prevent relegating these vulnerable populations to not receiving vaccination based on assumptions that vaccination acceptability would be low, the results of this study are promising, encouraging the implementation of vaccination not only during public health emergencies, but also as part of routine immunization catch-up programs in prisons. Improving vaccination coverage in adults residing in prisons reduces health inequities among marginalized populations.

## Figures and Tables

**Table 1:** Demographic characteristics (n = 1304).

	Prisoners *n* (%)	Prison Staff *n* (%)	*p*-Value	Total *n* (%)
	1016 (77.91)	288 (22.08)		1304 (100)
Sex			<0.001	
Male	914 (89.96)	147 (51.04)		1061 (81.36)
Female	102 (10.03)	141 (48.95)		243 (18.63)
Nationality			<0.001	
Spanish	941 (92.61)	286 (99.31)		1227 (94.10)
Non-Spanish	75 (7.38)	2 (0.69)		77 (5.9)
Cohabitation			<0.001	
Only	408 (40.15)	69 (23.95)		477 (36.57)
As a couple	608 (59.84)	219 (44.4)		827 (63.42)
Age (years)			<0.001	
18–<40	455 (44.78)	76 (26.38)		531 (40.72)
40–<60	464 (45.67)	207 (71.88)		671 (51.46)
>60	97 (9.55)	5 (1.74)		102 (7.82)
Children			0.007126	
No	301 (29.62)	110 (38.19)		411 (31.52)
Yes	715 (70.37)	178 (61.8)		893 (68.48)
Chronic disease			<0.001	
No	665 (65.45)	233 (80.90)		898 (68.86)
Yes	351 (34.54)	55 (19.09)		406 (31.13)
Tobacco use			<0.001	
No	254 (25.0)	272 (94.44)		526 (40.34)
Yes	762 (75.0)	16 (5.55)		778 (59.66)
Alcohol use			<0.001	
No	795 (78.24)	282 (97.91)		1077 (82.59)
Yes	221 (21.75)	6 (2.1)		227 (17.41)
Use of psychotropic drugs			<0.001	
No	678 (66.73)	285 (98.96)		963 (73.85)
Yes	338 (33.27)	3 (1.04)		341 (26.15)

**Table 2:** COVID-19 disease in prisoners and workers surveyed (*n* = 1304).

	Prisoners *n* (%)	Prison Staff *n* (%)	*p*-Value	Total *n* (%)
	1016 (77.91)	288 (22.08)		1304 (100)
Have you had COVID-19 disease?				
Yes	100 (9.84)	24 (8.33)	0.5113	124 (9.5)
No	916 (90.15)	264 (91.66)		1180 (90.5)
Have you had to quarantine for contact with a COVID-19 case?				
Yes	708 (69.68)	70 (24.30)	<0.001	778 (59.66)
No	308 (30.31)	218 (75.69)		526 (40.33)
Has a family member or friend died from COVID-19?				
Yes	192 (18.89)	53 (18.40)	0.9169	245 (18.78)
No	824 (81.10)	235 (81.59)		1059 (81.21)
Have you had any diagnostic tests, antigen tests or PCRs to detect SARS-CoV-2 at any time during this pandemic?				
Yes	685 (67.42)	150 (52.08)	<0.001	835 (64.03)
No	331 (32.57)	138 (47.91)		469 (35.96)
What were the results of the antigen test or PCR test to detect SARS-CoV-2?				
Positive	100 (14.59)	12 (8)	0.04384	112 (13.41)
Negative	585 (85.40)	138 (92)		723 (86.58)
Have you reduced your social interactions (visits, contacts with other groups, etc.) during the pandemic?				
Yes	330 (32.48)	285 (98.95)	<0.001	615 (47.16)
No	686 (67.51)	3 (1.05)		689 (52.84)

**Table 3:** COVID-19 vaccine acceptance and attitude toward vaccination (*n* = 1304).

	Prisoners *n* (%)	Prison Staff *n* (%)	*p*-Value	Total *n* (%)
	1016 (77.91)	288 (22.08)		1304 (100)
If the COVID-19 vaccine were available to you, would you get vaccinated?				
Yes	885 (87.10)	272 (94.44)	<0.001	1157 (88.72)
No	117 (11.51)	4 (1.38)		121 (9.2)
I would wait a few more months	6 (0.59)	7 (2.43)		13 (0.99)
I would wait for others to try it before	8 (0.78)	5 (1.73)		13 (0.99)
Would you vaccinate your children when the COVID-19 vaccine is available for them?				
Yes	659 (64.86)	224 (77.77)	<0.001	883 (67.71)
No	106 (10.43)	23 (7.98)		129 (9.89)
I would wait a few more months	92 (9.05)	25 (8.68)		117 (8.97)
I would wait for others to try it before	159 (15.65)	16 (5.55)		175 (13.42)
Would you recommend the COVID-19 vaccine?				
Yes	891 (87.70)	278 (96.53)	<0.001	1169 (89.64)
No	125 (12.30)	10 (3.47)		135 (10.35)
Do you think the benefit of getting vaccinated outweighs that of not getting vaccinated?				
Yes	879 (86.52)	278 (96.53)	<0.001	1157 (88.73)
No	137 (13.48)	10 (3.47)		147 (11.27)
Would it seem appropriate to be required to be vaccinated for certain activities?				
Yes	711 (69.98)	209 (72.57)	0.4368	920 (70.55)
No	305 (30.02)	79 (27.43)		384 (29.45)
Did you get the flu vaccine during the 2019–2020 vaccination campaign?				
Yes	135 (13.29)	160 (55.56)	<0.001	295 (22.62)
No	881 (86.71)	128 (44.44)		1009 (77.38)
Have you received the COVID-19 vaccine?				
Yes	988 (97.24)	256 (88.89)	<0.001	1244 (95.39)
No	28 (2.76)	32 (11.11)		60 (4.61)
If you have received it, what vaccine did you receive?				
Pfizer/BioNTech Comirnaty^®^	91 (9.21)	66 (25.78)	<0.001	157 (12.62)
Spikevax^®^ by Moderna	12 (1.21)	38 (14.84)		50 (4.01)
Vaxzevria^®^ by AstraZeneca	86 (8.70)	151 (58.98)		237 (19.05)
Jcovden^®^ Janssen	799 (80.87)	1 (0.39)		800 (64.30)

**Table 4:** Relative risk ratios from the multinomial logistic regression model and odds ratios from the logistic regression model.

Questions Related to COVID-19 Vaccine Acceptance									*p*-Value
If the COVID-19 Vaccine Were Available to You, Would You Get Vaccinated?									
		**No**	**Yes**		**I would wait a few more months**		**I would wait for others to try it before**		
			**RRR** **(CI95%)**	***p*-value**	**RRR (CI95%)**	***p*-value**	**RRR** **(CI95%)**	***p*-value**	
Sex	Female	Ref.	Ref.		Ref.		Ref.		0.0031
	Male	Ref.	0.33 (0.16–0.65)	0.0015	0.27 (0.06–1.15)	0.077	0.44 (0.08–2.31)	0.333	
Age	18–<40	Ref.	Ref.		Ref.		Ref.		<0.001
	40–<60	Ref.	1.31 (0.89–1.89)	0.17	0.44 (0.18–1.49)	0.19	0.61 (0.19–1.98)	0.41	
	>60	Ref.	8.73 ×$\;10^{13}$	0.00	7.2 × $\;10^{-5}$	NaN	7.2 × $\;10^{-5}$	0.00	
Chronic disease	No	Ref.	Ref.		Ref.		Ref.		0.0015
	Yes	Ref.	1.86 (1.18–2.94)	0.008	4.2 × $\;10^{-13}$	0.00	1.15 (0.29–4.51)	0.84	
Would you vaccinate your children when the COVID-19 vaccine is available for them?									
		**No**	**Yes**		**I would wait a few more months**		**I would wait for others to try it before**		
			**RRR (CI95%)**	***p*-value**	**RRR (CI95%)**	***p*-value**	**RRR (CI95%)**	***p*-value**	
Sex	Female	Ref.	Ref.		Ref.		Ref.		<0.001
	Male	Ref.	0.83 (0.49–1.39)	0.48	0.21 (0.11–0.38)	<0.001	1.42 (0.72–2.8)	0.32	
Age	18–<40	Ref.	Ref.		Ref.		Ref.		<0.001
	40–<60	Ref.	1.28 (0.88–1.88)	0.19	0.29 (0.17–0.5)	<0.001	0.48 (0.3–0.7)	0.002	
	>60	Ref.	7928.78 (6.1 × $\;10^{-14}$–1.1 × $\;10^{21}$)	0.66	0.28 (2.3 × $\;10^{-32}$–3.41 × $\;10^{30}$)	0.97	0.38 (1.9 × $\;10^{-27}$–7.5 × $\;10^{25}$)	0.97	
Chronic disease	No	Ref.	Ref.		Ref.		Ref.		<0.001
	Yes	Ref.	2.89 (1.77–4.71)	<0.001	1.19 (0.61–2.3)	0.6	1.78 (0.99–3.17)	0.05	
Would you recommend the COVID-19 vaccine?									
		**No**	**Yes**						
			**OR (CI95%)**	***p*-value**					
Sex	Female	Ref.	Ref.						0.0014
	Male	Ref.	0.36 (0.18–0.65)	0.0015					
Age	18–<40	Ref.	Ref.						<0.001
	40–<60	Ref.	1.42 (0.98–2.03)	0.06					
	>60	Ref.	6.5 × $\;10^{6}$	0.97					
Chronic disease	No	Ref.	Ref.						0.0043
	Yes	Ref.	1.92 (1.26–3.03)	0.0036					
Do you think the benefit of getting vaccinated outweighs that of not getting vaccinated?									
		**No**	**Yes**						
			**OR (CI95%)**	***p*-value**					
Sex	Female	Ref.	Ref.						<0.001
	Male	Ref.	0.36 (0.18–0.63)	<0.001					
Age	18–<40	Ref.	Ref.						<0.001
	40–<60	Ref.	1.76 (1.24–2.5)	0.0015					
	>60	Ref.	7.88 × $\;10^{6}$	0.97					
Chronic disease	No	Ref.	Ref.						<0.001
	Yes	Ref.	2.16 (1.42–3.41)	<0.001					
Would it seem appropriate to be required to be vaccinated for certain activities?									
		**No**	**Yes**						
			**OR (CI95%)**	***p*-value**					
Sex	Female	Ref.	Ref.						0.042
	Male	Ref.	0.71 (0.51–0.97)	0.035					
Age	18–<40	Ref.	Ref.						<0.001
	40–<60	Ref.	0.82 (0.64–1.04)	0.1					
	>60	Ref.	6.54 (3.04–17.02)	<0.001					
Chronic disease	No	Ref.	Ref.						0.06
	Yes	Ref.	1.3 (0.99–1.68)	0.06					
Did you get the flu vaccine during the 2019–2020 vaccination campaign?									
		**No**	**Yes**						
			**OR (CI95%)**	***p*-value**					
Sex	Female	Ref.	Ref.						<0.001
	Male	Ref.	0.42 (0.31–0.56)	<0.001					
Age	18–<40	Ref.	Ref.						<0.001
	40–<60	Ref.	2.8 (2.04–3.88)	<0.001					
	>60	Ref.	12.4 (7.71–20.21)	<0.001					
Chronic disease	No	Ref.	Ref.						<0.001
	Yes	Ref.	3.04 (2.33–3.98)	<0.001					

**Table 5:** Beliefs and occurrence of adverse effects following COVID-19 vaccine.

	Prisoners *n* (%)	Prison Staff *n* (%)	*p*-Value	Total *n* (%)
	1016 (77.91)	288 (22.08)		1304 (100)
Do they produce more adverse effects than other vaccines?				
Yes	171 (16.83)	96 (33.33)	<0.001	267 (20.47)
No	845 (83.17)	192 (66.67)		1037 (79.52)
Are there any unknown adverse effects from the COVID-19 vaccine?				
Yes	722 (71.06)	167 (57.99)	<0.001	889 (68.17)
No	294 (28.94)	121 (42.01)		415 (31.82)
Does the accelerated pace of vaccine production reduce its safety?				
Yes	376 (37.01)	131 (45.49)	0.01119	507 (38.88)
No	640 (62.99)	157 (54.51)		797 (61.11)
Do you trust the recommendations of the health authorities?				
Yes	817 (80.41)	250 (86.81)	0.01656	1067 (81.82)
No	199 (19.59)	38 (13.19)		237 (18.17)
After vaccination, have you experienced adverse effects? (*n* = 1244)				
Yes	519 (52.53)	179 (69.92)	<0.001	698 (56.11)
No	469 (47.47)	77 (30.08)		546 (43.89)
Which side effects have you experienced? (*n* = 698)				
Pain site of administration	350 (67.43)	138 (77.09)	<0.001	488 (69.91)
Fatigue	270 (52.02)	119 (66.48)		389 (55.73)
Fever	165 (31.79)	97 (54.10)		262 (37.53)
Generalized muscle aches	304 (58.57)	111 (62.01)		415 (59.45)
Headache	279 (53.75)	88 (49.16)		367 (52.57)
Chills	303 (58.38)	99 (55.30)		402 (57.59)
Inflammation	11 (2.11)	13 (7.26)		24 (3.43)
Vomiting	17 (3.2)	6 (3.35)		23 (3.29)

## Data Availability

Data is unavailable due to privacy or ethical restrictions.
